# Jellyfish Polysaccharides for Wound Healing Applications

**DOI:** 10.3390/ijms231911491

**Published:** 2022-09-29

**Authors:** Chiara Migone, Noemi Scacciati, Brunella Grassiri, Marinella De Leo, Alessandra Braca, Dario Puppi, Ylenia Zambito, Anna Maria Piras

**Affiliations:** 1 Department of Pharmacy, University of Pisa, Via Bonanno 33, 56126 Pisa, Italy; 2 Centre for Instrument Sharing of University of Pisa (CISUP), University of Pisa, Lungarno Pacinotti 43, 56126 Pisa, Italy; 3 Department of Chemistry and Industrial Chemistry, INSTM-University of Pisa Research Units (UdR Pisa), University of Pisa, Via Moruzzi 13, 56124 Pisa, Italy

**Keywords:** jellyfish, polysaccharides, glycosaminoglycan, wound healing

## Abstract

Jellyfishes are considered a new potential resource in food, pharmaceutical and biomedical industries. In these latter cases, they are studied as source of active principles but are also exploited to produce marine collagen. In the present work, jellyfish skin polysaccharides (JSP) with glycosaminoglycan (GAG) features were extracted from *Rhizostoma pulmo*, a main blooming species of Mediterranean Sea, massively augmented by climate leaded “jellyfishication” of the sea. Two main fractions of *R. pulmo* JSP (RP-JSPs) were isolated and characterized, namely a neutral fraction (RP-JSP1) and a sulphate rich, negatively charged fraction (RP-JSP2). The two fractions have average molecular weights of 121 kDa and 590 kDa, respectively. Their sugar composition was evaluated through LC-MS analysis and the result confirmed the presence of typical GAG saccharides, such as glucose, galactose, glucosamine and galactosamine. Their use as promoters of wound healing was evaluated through in vitro scratch assay on murine fibroblast cell line (BALB/3T3 clone A31) and human keratinocytes (HaCaT). Both RP-JSPs demonstrated an effective confluency rate activity leading to 80% of scratch repair in two days, promoting both cell migration and proliferation. Additionally, RP-JSPs exerted a substantial protection from oxidative stress, resulting in improved viability of treated fibroblasts exposed to H_2_O_2_. The isolated GAG-like polysaccharides appear promising as functional component for biomedical skin treatments, as well as for future exploitation as pharmaceutical excipients.

## 1. Introduction

Polysaccharides are fundamental components of living organisms and are ubiquitary in animals, plants, microorganisms, and viruses [1]. The polysaccharides, albeit with a simple three-dimensional structure, can be composed of a wide variety of saccharide residues, organized in the form of homopolysaccharide or heteropolysaccharides, arranged either linearly or in branched structures [2]. This variety of composition confers several functions to the natural polysaccharides, i.e., energy reserve, tissue structural component, bioactivity. On the other hand, polysaccharides find a wide application in pharmaceutical technology, food and cosmetic applications, not only for their gelling capability, adsorbent, disaggregation and binding features and but also as co-adjuvants in immunomodulatory, antioxidant, antimicrobial, antitumor, anti-glycaemic purposes [3,4].

Marine sources are attracting increasing interest, for being rich of active molecules and biomaterials for pharmaceutical and biomedical applications [5]. There are several examples of marine derived macromolecules with applications in the pharmaceutical field and thanks to their structural similarity to the component of the extracellular matrix (ECM), they have been studied and commercially applied into tissue regeneration products, as well as safe pharmaceutical excipients [6].

As example, compounds derived from marine algae such as fucoidans and ulvans, sulphated polysaccharides present in the cell walls of algae, appear promising in wound healing and skin regeneration applications [7,8]. Furthermore, the use of different types of marine macromolecules, such as collagen or chitosan, in engineered skin tissues constructs [9] as well as in spray patches [10], confirmed that macromolecules of marine origin possess unique characteristic for applications in the biomedical field [11].

Marine polysaccharides (e.g., alginate, ulvans, chitin, and chondroitin sulphate) are widely applied also for bio-adhesive drug delivery formulations, due to their biocompatibility (generally recognized as safe—GRAS materials), and intrinsic bioactivity such as mucoadhesion properties, anti-inflammatory effects and wound healing promotions [12]. Several of these features are related to their natural origin as glycosaminoglycans (GAG), the polysaccharide sidechain of mucopolysaccharides (proteoglycans). Marine derived biomasses, such as cartilages of octopus, squid, shark, and bones of monkfish, codfish, salmon, tuna, and sturgeon are presently exploited for the extraction of GAGs [13]. Jellyfish may represent a new biomass. Jellyfish are mainly used in Asian countries, and recently in western countries, for the extraction of collagen and to prepare functional food products. However, the “jellyfishication” of Mediterranean marine ecosystems, mainly due to climate changes and to the lack of natural predators for jellyfish, has not a present reflection on jellyfish consumption in western countries. As consequence, it causes a serious economic impact on costal economies (tourism, fishing, fish farming) [14]. Similar to other marine polysaccharides, Jellyfish Skin Polysaccharides (JSP) are emerging as interesting biomaterials with immunomodulatory effect [15,16] and anti-inflammatory properties when tested as dietary supplement in murine inflammatory bowel disease (IBD) model [17]. Until now, only JSP from Pacific species (i.e., *Rhopilema esculetum*) have been investigated, here we propose the evaluation of JSP from a blooming species of Mediterranean Sea, *Rhizostoma pulmo*. *R. pulmo* and *Pelagia noctiluca* are the two main blooming species in the Mediterranean area, but only *R. pulmo* is a non-toxic edible species [18,19]. *R. pulmo* is an endemic Mediterranean jellyfish widely distributed across the sea from Spain to Marmara Sea and in the Black Sea. In the last years, the spread of *R. pulmo* has been increased along the Italian coasts and in the Ionian Sea, where it has been present regularly since 2005, acquiring high abundances from July to October. Recently, a remarkable *R. pulmo* outbreak (over 48,000 ind/km^2^ and a biomass assessment of ~300 t/km^2^) has been estimated along the southwestern shores of the Gulf of Taranto using an ultra-light aerial survey [20]. On the European coasts, jellyfish masses cause serious damage to aquaculture and mariculture companies, as well as blocking the cooling systems of power plants near the coast. Jellyfish blooms damage fish farms and tourism economics, impair recruitment in fish population of commercial interest, and are causing detrimental impacts to many coastal ecosystems [21,22]. The exploitation of blooming jellyfish is seen as an opportunity with direct economic impact on costal economies, as well as a way to promote the marine biodiversity, compromised by jellyfishication. In this view, we extracted JSP from Mediterranean *R. pulmo* specimens. Two main high molecular weight fractions were distinguished, showing glucosaminoglycan-like features, good thermal stability, and demonstrating both cell migration effects and protection versus oxidative stress. Their future exploitation in wound healing applications is foresees.

## 2. Results 

### 2.1. Characteristics of the Extracted Polysaccharides

The polysaccharides extraction procedure was carried out adapting the conditions described for *Rhopilema esculetum* species [15,16] to *R. pulmo*. Starting from 1.9 kg of fresh jellyfish, RP-CrudeJSP (3.05 g) was obtained by extraction in warm water, precipitation in ethanol and deproteinization through Sevag method. An aliquot of the crude fraction (2.5 g) was further purified in DEAE Sepharose anionic exchange column, obtaining two separate fractions: RP-JSP1 from water elution and RP-JSP2 from NaCl elution (Figure S1). After desalting, 244.6 mg and 190.6 mg of RP-JSP1 and RP-JSP2 were collected, respectively. 

**Figure 1:**
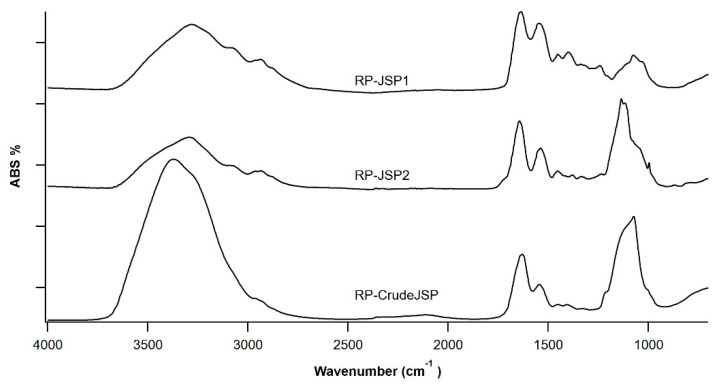
FT-IR spectroscopy of polysaccharides from *R. pulmo* jellyfish: RP-JSP1, RP-JSP2, and RP-CrudeJSP.

RP-CrudeJSP, RP-JSP1 and RP-JSP2 were characterized through ATR-FT-IR spectroscopy (Figure 1). All samples show a broad stretching vibration peak of O-H and N-H moieties between 3500–3200 cm^−1^ and the stretching of CH_2_ and CH_3_ groups in the range 2940–2870 cm^−1^. The presence of functional moieties, such as amide, amine and carboxyl, is revealed by the two peaks at 1633 cm^−1^ and 1540 cm^−1^, typical of galactosamine, glucosamine and glucuronic residues. These peaks are overlapping for RP-CrudeJSP and RP-JSP1, only a slight shifting is observed for RP-JSP2, where the two signals are at 1645 cm^−1^ and 1539 cm^−1^, respectively. In RP-JSP2 spectra, a shoulder at higher wavenumber (1715 cm^−1^) suggests for uronic acid presence. The signal at 1400 cm^−1^ is also suggesting for the presence of uronic acid [23]. At 1030–1100 cm^−1^ the bands due to glycosidic bonds of sugar rings and –OH are present in all samples. For RP-JSP2, the C–O–S band at 860 cm^−1^, typical of sulphated polysaccharides [24], is displayed. Concerning the ^1^H NMR spectra of RP-CrudeJSP, RP-JSP1 and RP-JSP2 fractions (Figure S2), they are similar to literature spectra of JSP from other jellyfish species [16]. At 1.0–2.0 ppm the signals deriving from methylene groups and at 2.0 ppm the possible assignment to acetyl groups owned from *N*-acetyl-glucosamine/galactosamine. Among 3.4 and 4.0 ppm the signals due to the sugar rings, including protons in alpha to sulphate moieties at 3.13 ppm, 3.43 ppm and 3.61 ppm, in case of RP-JSP2 fraction [25]. The anomeric peaks are present at 4.5–5.3 ppm. Peaks at 6.5–7.5 ppm can be assigned to residual aromatic amino acids covalently bound to the polysaccharidic fractions. Indeed, those residual protein fractions increase the complexity of the acquired ^1^H NMR [16]. 

The molecular weight (Mw) of RP-JSP1 and RP-JSP2 was determined by Deybe Plot analysis, whereas the amount of residual protein was determined by bicinchoninic acid analysis (BCA) and the quantification of sulphate moieties was performed by BaCl_2_-gelatine method. The main characteristics of the collected fractions are reported in Table 1. The Mw of RP-CrudeJSP was not determined due to the high polydispersity of the sample [15], which makes it unsuited for a precise MW calculation. Concerning the purified fractions, RP-JSP1 and RP-JSP2, both have a medium high molecular weight, compatible with common GAG such as hyaluronic acid (in the range of 10^4^–10^6^ Da). As expected, RP-JSP2 has the highest content of sulphate moieties, similar to that of sulphated GAG such as chondroitin sulphate (12–20%wt). Accordingly, the RP-CrudeJSP fraction contains a high sulphate content as well. 

**Figure 2:**
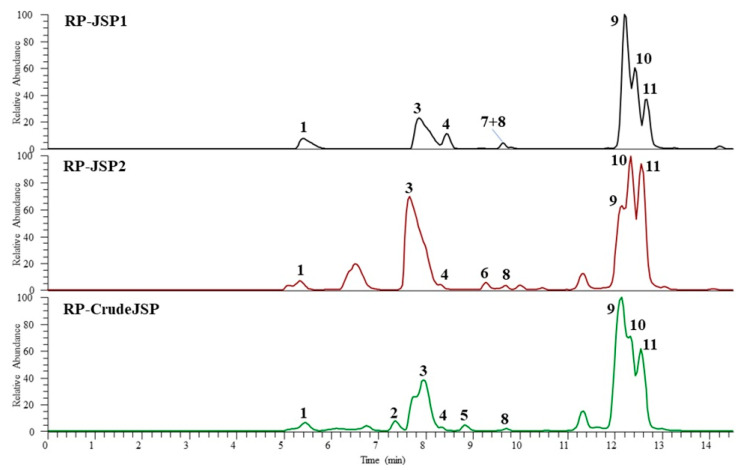
UHPLC-HR-ESI-Orbitrap/MS profiles (negative ionization mode) of hydrolyzed 1-phenyl-3-methyl-5-pyrazolone (PMP) derivatives of polysaccharides from *R. pulmo* jellyfish: RP-CrudeJSP (green), RP-JSP1 (black), and RP-JSP2 (red) samples.

In order to define the monosaccharide composition of RP-CrudeJSP, RP-JSP1, and RP-JSP2 fractions, the samples were analyzed through UHPLC-MS after hydrolysis and derivatization with 1-phenyl-3-methyl-5-pyrazolone (PMP). The chromatograms obtained operating in negative ESI mode are shown in Figure 2. 

The monosaccharide identification was performed by comparison of chromatographic data, full and fragmentation HR mass spectra (Table 2) with those obtained by injection of reference standards (chitosan, starch, and chondroitin sulphate), hydrolyzed and PMP derivatized in the same conditions of the studied samples. The results showed that all fractions are characterized by the presence of five main components, identified as glucosamine (peak 1), galactosamine (peak 3), glucose (peak 9), galactose (peak 10), and an unidentified pentose (peak 11). In addition, two more hexoses (peaks 2 and 4) were detected in the RP-CrudeJSP and all fractions, respectively. Peaks 5, 6, and 8, showing the same ESI full and fragmentation spectra of peak 11, were attributed to pentose residues. Finally, a deoxyhexose (peak 7) was revealed in the RP-JSP1. In agreement with those findings, previous results from polysaccharide analyses of *Rhopilema esculentum* JSP fraction evidenced the presence of glucose, galactose, mannose, and arabinose [15,16,17].

### 2.2. Thermal Characterization

**Figure 3:**
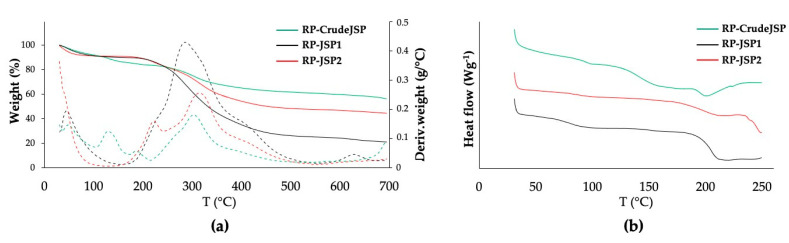
Thermal properties assessment of RP-CrudeJSP (green), RP-JSP1 (black) and RP-JSP2 (red). (**a**) TGA analysis: weight loss (full line) and derivative weight (dashed line) vs. temperature; (**b**) DSC analysis: heat flow *vs*. temperature curves of the analyzed samples relevant to the second heating scan.

Materials thermal properties were evaluated by means of thermogravimetric analysis (TGA) and differential scanning calorimetry (DSC). Representative TGA thermograms of the three samples are reported in Figure 3a and DSC thermograms in Figure 3b. All three samples were characterized by an initial weight loss in the temperature range 25–120 °C mainly related to evaporation of adsorbed water molecules, as well as a main degradation step centered in the temperature range 280–320 °C likely related to polysaccharide chain decomposition [26] (Table 3).

RP-JSP2 and RP-CrudeJSP thermograms were also characterized by other derivative weight peaks at lower temperatures (140 °C and 210 °C, respectively). Differences in residual weight at 700 °C among the different samples were evident. In particular, RP-JSP1 had a residual weight of around 20%, RP-JSP2 of 45% and RP-CrudeJSP of 56%. Such differences could be related to a different concentration of inorganic/mineral phase and counter-ions of the salt forms. Concerning the DSC thermographs, RP-JSP1 had a Tg (82 °C) lower than RP-JSP2 (89 °C) and RP-CrudeJSP (94 °C) (Table 3). 

### 2.3. Biological Evaluation of Extracted Polysaccharides for Wound Healing Application

#### 2.3.1. Scratch Test and Protective Effect from Oxidative Stress on Fibroblast BALB/3T3 Cells

According to ISO 10993-5 [27], fibroblasts BALB/3T3 clone A31 were selected in the present study for a preliminary biological investigation and because of their involvement in wound healing [28]. In order to select the optimal concentration for the Scratch test assay, the cytotoxicity of RP-CrudeJSP, RP-JSP1 and RP-JSP2 was investigated. The results are reported in Figure S3. For all tested samples, cell viability was reducing with increasing sample concentration. In particular, the data obtained after 24 h of incubation exhibited a good cell viability in the concentration range 0.015–0.125 mg/mL for all the tested samples showing cell viability values more than 90%. At higher concentrations cell viability decreased maintaining percentages of 60%, though (Figure S3a). Regarding the data acquired after 48h incubation, cell proliferation decreased with increasing sample concentrations (Figure S3b). The graph profile showed more than 80% of cell proliferation values for all the samples at 0.01 mg/mL, whereas at 0.5 mg/mL the percentages decreased to 40–50% values. The data confirms the good cytocompatibility described for other JSP on macrophages cell line [16].

**Figure 4:**
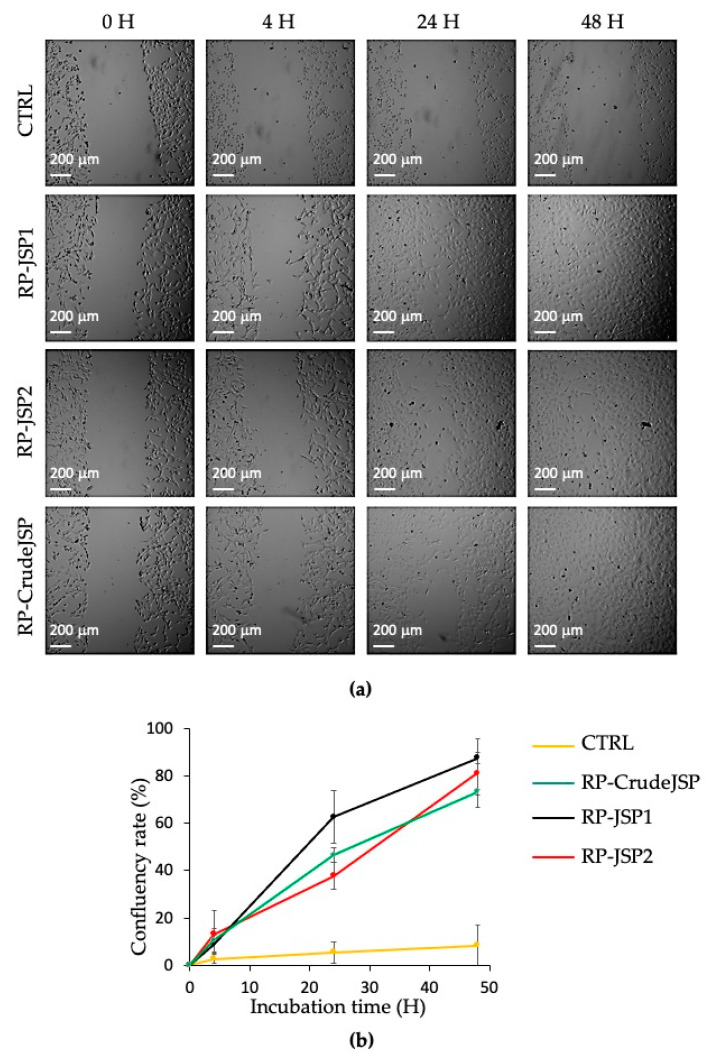
Scratch test on BALB/3T3 clone A31 murine embryonic fibroblast cell monolayers treated with *R.pulmo* polysaccharides (RP-CrudeJSP, RP-JSP1, and RP-JSP2). Control consists in untreated cells (CTRL). (**a**) Representative micrographs (4× magnification) of treated and control monolayers; (**b**) confluency rate calculated as percentage of scratch closure in respect to initial scratch area.

The scratch test assay is a well-developed method based on a mechanical scratch wounding of confluent monolayers. The test simplifies the wound healing process, allowing for cell migration at the wound rims and layer regeneration by proliferation [10,29]. The technique involves a scratch made with a pipette tip to create an incision-like gap. Low serum concentrations (1% FBS) in the cell medium were used to suppress the cell proliferation in wound healing tests [30]. To evaluate the effect of RP-CrudeJSP, RP-JSP1 and RP-JSP2 on the migration of fibroblasts, a scratch test was performed on a cellular monolayer and its ability to heal after 4, 24 and 48 h was investigated (Figure 4a). All the samples provided accelerated cell migration and confluency rate, respect of the control (Figure 4b). More specifically, while the control confluency rate percentage after 48 h was of 3%, RP-JSP1, RP-JSP2 and RP-CrudeJSP reached about 80–90% of scratch closure. No statistical difference between the three samples was detected. 

**Figure 5:**
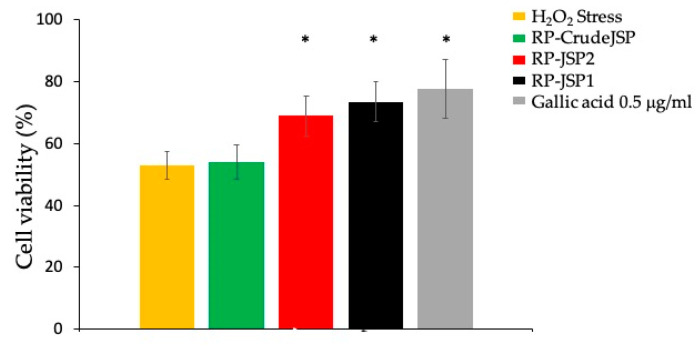
Protective effects from H_2_O_2_-induced oxidative stress. Histograms representing BALB/3T3 cell viability after 2 h of pre-treatment with *R. pulmo* polysaccharides (RP-CrudeJSP, RP-JSP2, RP-JSP1) or 0.5 μg/mL of gallic acid (reference sample), followed by 1500 μM H_2_O_2_ for 1 h. Means ± SD (n = 8). * *p* < 0.01 vs. H_2_O_2_ Stress.

Preliminary assessment of the absence of readily reactive antioxidant species in the extracted JSP was performed by *Folin-Ciocalteau* method [31] (Supplementary File). Afterwards, the protection of the JSP toward oxidative stress was investigated. Cells were pre-treated for 2 h with 0.125 mg/mL of RP-CrudeJSP, RP-JSP1 and RP-JSP2, then the oxidative damage was inflicted with H_2_O_2_ addition. As indicated in Figure 5, the treatment with H_2_O_2_ reduced cell viability to 53% (H_2_O_2_ Stress—control), compared to the reference sample (not treated and not stressed). Concerning the JSP samples, the results obtained demonstrated a significant protective activity (*p* < 0.01) from H_2_O_2_ oxidative damage when cells were pre-treated with the purified RP-JSP1 and RP-JSP2 fractions (cell viability values of 74% and 69%, respectively). The cells pre-treated with RP-CrudeJSP sample showed cell viability values of 54%, analogous to the stressed control, showing no protection from the H_2_O_2_ treatment. 

#### 2.3.2. Keratinocytes Scratch Wound Healing Assay

Preliminary assessment of RP-JSP1 and RP-JSP2 cytotoxicity on HaCat cells by the WST-1 assay are reported in Figure S4. After 24 h of incubation, no cytotoxicity effect was observed for all samples (Figure S4a). Differently, the cell proliferation at 48 h was reducing with increasing sample concentrations (Figure S4b). In particular, for concentrations ranging from 0.1 to 0.12 mg/mL, RP-JSP1 fraction showed cell proliferation around 70–80%, whereas at higher concentrations (0.25–2 mg/mL) cell proliferation values were round 50–60%. In addition, in case of HaCat cell lines, the good biocompatibility of the RP-JSPs fractions was confirmed.

**Figure 6:**
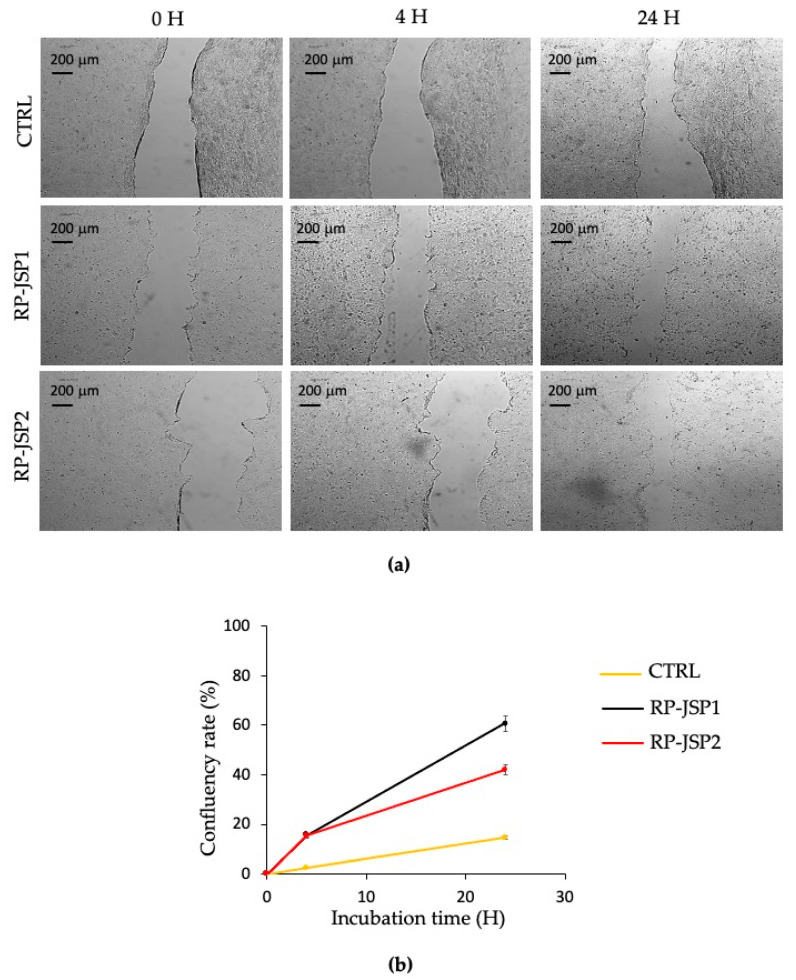
Scratch test on HaCat human keratinocytes cell monolayers treated with RP-JSP1 and RP-JSP2. Control consists in untreated cells. (**a**) Representative micrographs (4× magnification) of treated and control monolayers; (**b**) confluency rate calculated as percentage of scratch closure in respect to the initial scratch area.

The effects of RP-JSP1 and RP-JSP2 fractions on keratinocytes migration were evaluated by means of a scratch test performed on a cell monolayer, monitoring the ability to heal for 24 h (Figure 6). Compared to the controls, which showed a 15% of confluency rate percentages, RP-JSP1 and RP-JSP2 fractions reached percentage values of 60% and 43%, respectively. In addition, for keratinocytes, it is observed a faster trend for RP-JSP1 fraction.

## 3. Discussion

Due to their high abundance and high regenerative power, Cnidarians can be considered a new source of pharmaceuticals, nutraceuticals and food/feed compounds [32,33]. In the present work, polysaccharides from *R. pulmo* were extracted adapting the protocol conditions described for the *R. esculetum* species [15]. The extracted RP-CrudeJSP was subjected to a further purification process through an anion exchange chromatographic column, distinguishing two main fractions, namely RP-JSP1 and RP-JSP2, with the latter displaying net negatively charged moieties (Figure S1). All samples have a similar monosaccharide compositions and residual protein contents (Table 1 and Table 2). Differently from other reports focusing on a not charged fraction [15,16,17], in this paper the not charged (RP-JSP1) and a negatively charged (RP-JSP2) fractions were isolated and both characterized. RP-JSP2 has a higher molecular weight and a high content of sulphate moieties (Table 1). Both samples have GAG-like features, as evidenced by the physical-chemical characterizations (Figure 1, Figure 2 and Figure S2), resembling hyaluronic acid and chondroitin sulphate polysaccharides [13]. Additionally, the pristine RP-CrudeJSP was also tested, displaying cytocompatibility and wound healing promotion, such as the purified RP-JSP1 and RP-JSP2 components (Figure 4, Figure 6, Figure S3 and Figure S4). By comparing the obtained protein concentrations, it was observed that the percentage of protein content was about 20% for all the samples, regardless the further step of ion exchange chromatography, performed for separating RP-JSP1 and RP-JSP2. The Sevag method used for the deproteinization consists of a physical purification of polysaccharides, which is effective for the removal of proteins but less successful in removing protein residues strongly associated or covalently bound to the polysaccharidic chains [34]. The percentage of protein found in all the fractions confirmed the presence of glycoproteins or complex protein/polysaccharide systems, in agreement with the proteoglycan nature of GAGs. Indeed, sulfated glycosaminoglycans (GAGs) are complex polysaccharides, which are covalently bound to protein cores to form proteoglycans [35]. Additionally, the association of enzymatic and Sevag deproteinization treatments to JSP extraction has been already evaluated. Since some residual protein content was still present [15], only Sevag treatment was here applied to simplify the procedure. 

Regarding thermal characterization (Figure 3 and Table 3), data are in line with thermal degradation properties of chondroitin sulphate and other polysaccharides of marine origin [36]. According to Vasconcelos Oliveira et al. [37] the destruction of chondroitin sulphate begun at approximately at 250 °C and can rise to 300 °C depending on the saccharide composition. The high degradation temperature of the RP-JSPs indicate their suitability for further use into biomedical devices, encountering elevated manufacturing temperatures, such as spray drying technique [38]. Important differences were observed for the residual weight at 700 °C, likely due to the different concentration of inorganic/mineral phase and counterions of the salt forms [39]. 

Polysaccharide-based materials are widely used in wound dressings [11]. Wound healing evaluation was performed by scratch test. Cell migration was evaluated with respect to time, since a rapid and a functional wound closure is the primary wound healing target [30]. In the context of skin wounds, injury healing following hemostasis occurs in three overlapping stages: inflammation, proliferation, and remodeling. Fibroblasts have important roles in all three steps, acting a key function in the deposition of extracellular matrix components, wound contraction and remodeling [40]. By analyzing the area of the captured images (Figure 4), it was observed that the scratch closure rate of untreated cells (CTRL) was slower than those of RP-JSPs samples. Comparing the fibroblasts images, it was noticed the cell migration towards the center of the scratch to repair the tear. At time zero the rims were clear, after 24 h the cell migration toward the center was appreciable, and at 48 h the monolayers appeared completely confluent. Hence, RP-CrudeJSP, RP-JSP1 and RP-JSP2 exhibited a positive effect on cell migration compared to control samples. Despite the treatments with the RP-JSPs were statistically equal, it is interesting to notice that the trend observed for RP-CrudeJSP sample resulted as the average of those of its components RP-JSP1 and RP-JSP2.

Despite showing similar biocompatibility and wound healing stimulation, the three tested samples resulted in different antioxidant activities. Only the not purified RP-CrudeJSP sample did not show any protective effect at the tested concentrations (Figure 5). However, it cannot be excluded that higher concentrations could be effective. Several mechanisms have been associated to the antioxidant effect of GAGs. The most supported relates with a chelating action toward metal ions [41], the presence of functional protein residues [32,42], and the stimulation of biochemical pathways [43,44]. Concerning RP-JSP1 and RP-JSP2, the observed antioxidant effect could be related to the already mentioned mechanisms, since the performed Folin-Ciocâlteu evaluations excluded the presence of any readily reactive polyphenols or sugars. Nevertheless, the acquired data, excluded a simple stimulation of cell proliferation because of the short incubation time adopted for cell treatments (2 h). Such observation is supported by the performed cell cytotoxicity and scratch test studies, showing no significant differences between RP-CrudeJSP, RP-JSP1 and RP-JSP2 samples. 

Thanks to the promising results obtained by RP-JSP1 and RP-JSP2 in the protection against oxidative stress, both samples were tested on HaCaT cell line, selected as a substitute for normal human keratinocytes [45]. For wound healing purposes, fibroblast BALB/3T3 cells and HaCat cells are used as representative cell lines in the setting of skin injury [46,47]. Also in case of HaCaT cells, the treatments with either RP-JSP1 or RP-JSP2 promoted cell migration over time (Figure 6). After 24 h, cells had passed the tear margins and tended to cover the center of the scratch. These data were in accordance with the results described for polysaccharides extracted from *Gracilaria lemaneiformis* and *Auricularia auricula-judae* [48,49]. 

Taking together the reported findings and the literature data on the immunomodulating effect of JSP [15,16,17]; RP-JSPs appear as promising materials for further in vivo evaluation in wound healing, where inflammatory modulation, antioxidant action and cell migration have all key roles in the healing process [11,50]. 

## 4. Materials and Methods

### 4.1. Materials

*Rhizostoma pulmo* jellyfish was provided by Gionata Argelli, Liuba fishing boat, as waste material from offshore fishing in the Mediterranean Sea in front of Tuscany (Italy). Ethanol 99.8%, toluene 99.7%, acetone, ammonia 7 N in methanol, magnesium sulphate, sodium chloride, sodium carbonate, gallic acid, barium chloride, trifluoroacetic acid (TFA), 1-phenyl-3-methyl-5-pyrazolone (PMP) were obtained from Sigma-Aldrich (St. Louis, MO, USA). 1-butanol 99.5% was obtained from EMSURE^®^, chloroform 99.5% and hydrochloric acid 99.0–99.4% were purchased from Honeywell (Charlotte, NC, USA). Glacial acetic acid 100% was obtained from VWR chemicals (Milan, Italy). Bicinchoninic acid assay was purchased from Merck (St. Louis, MO, USA). Dehydrated water (D_2_O) 99.9% was obtained from Deutero GmbH (Kastellaun, Germany). Chitosan and starch were bought from Merck (St. Louis, MO, USA), while chondroitin sulphate was purchased from A.C.E.F (Piacenza, Italy). UHPLC grade methanol, formic acid, and water were purchased from Romil-Deltek (Pozzuoli, Italy).

### 4.2. Extraction and Purification of Polysaccharides 

The extraction of polysaccharides was performed according to Zhang H.L. et al. [15]. Briefly, *R. pulmo* was removed from tentacles, washed and weighed (1900 g). The sample were ground in a blender and mixed with deionized water in ratio 1:7.5 (kg/L). The mixture was maintained for 4 h at 95 °C under stirring and then was cooled at room temperature and centrifugated at 10,000 rpm for 15 min. The supernatant was collected and concentrated with rotary evaporator and next mixed with ethanol 80% (*v*/*v*) in ratio 1:4 and kept at 4 °C overnight. The precipitated polysaccharide fraction was recovered by 0.45 μm filtration under vacuum and dried in stove. The material was deproteinized by Sevag method [51]. The pellets were dissolved under energetic stirring in 500 mL of deionized water at 40 °C for 1 h. Sevag reagent (1-butanol/chloroform = 1/4) was added in ratio 5:2 to the solution and maintained under vigorous stirring for 1 h. The mixture was centrifugated at 6000 rpm for 20 min and three different layers were obtained: the top layer was collected and the process repeated 7 times until the complete disappearance of the middle layer. The solution was lyophilized to give crude jellyfish polysaccharide sample (RP-CrudeJSP). RP-CrudeJSP was further purified on DEAE Sepharose column, eluting a neutral fraction with water and a negatively charged fraction with 0.3 M NaCl. Elution was monitored by UV measurements at 210 nm. Both fractions were dialyzed (Float-A-Lyzer^®^G2, MWCO 1000 membrane) against water for 2 days and freeze-dried. RP-JSP1 (water eluted) and RP-JSP2 (NaCl 0.3M eluted) were stored in the desiccator until further use. 

### 4.3. Instruments 

Attenuated Total Reflection Fourier Transform Infrared (ATR-FTIR) spectroscopy was performed (Cary 660 series FT-IR, Agilent Techologies, Santa Clara, CA, USA). RP-CrudeJSP, RP-JSP1 and RP-JSP2 fraction were analyzed in % of absorbance, between 600–4000 cm^−1^ with resolution of 4 cm^−1^ and 32 number of scans.

^1^H NMR spectra were acquired on Bruker Ultrashield™ spectrometer working at 400 MHz for ^1^H, at 25 ± 0.1 °C on 2%wt, D_2_O samples. 

### 4.4. Molecular Weight Determination

The molecular weights of RP-JSP1 and RP-JSP2 were evaluated by building a Debye plot (Zetasizer, Nano series Malvern) based on the variation in scattering intensity given by the change of polymer concentration in solution. The weight average molecular weight was calculated according to:

KC/Ra = 1/M + 2A2C(1)

where K is optical constant, R_a_ is Rayleigh relationship, M is the weighted average molecular weight, A_2_ is virial coefficient and C is the concentration of the macromolecule in solution. Clear water solutions of either RP-JSP1 or RP-JSP2 were prepared in milliQ water (0.2–2.0 mg/mL) and 0.22 μm filter. The analysis was performed in glass cuvettes at 25 °C, equilibration time of 2 min and 12 interactions of 10 s each. Toluene was used as reference and the differential increase value of refractive index as function of concentration (dn/dc) was set at 0.150 mL/g (average value for polysaccharides [52]. 

### 4.5. Determination of Protein Content 

Bicinchoninic acid (BCA) assay was used to determine the residual protein fraction. Samples were prepared in NaCl 0.3M at a final concentration of 0.5 mg/mL. Bovine serum albumin (BSA) was used as standard for the calibration curve (2–100 μg/mL; R^2^ = 0.9994). 

### 4.6. Determination of Sulphate Groups’ Content

Sulphate moieties were quantified by gelatin—BaCl_2_ method [53]. Briefly, BaCl_2_-gelatin reagent was prepared by dispersing 25 mg of gelatin in 5 mL of water at 60–70 °C, then kept at 4 °C overnight. BaCl_2_ (25 mg) was added and left under stirring at room temperature for 3 h, followed by 1.2 μm filtration. Samples of 5 mg/mL were prepared in HCl 1 N and hydrolyzed at 100°C for 5 h. Then 40 μL of solution was mixed with 760 μL of TFA 4% (*p*/*v*) and 200 μL of BaCl_2_-gelatin reagent. After 20 min, the absorption was evaluated at λ 395 nm normalized on λ 500 nm. Standard samples of MgSO_4_ (0.1–2.0 mg/mL, R^2^ = 0.994) were used as reference. 

### 4.7. Monosaccharide Characterization 

#### 4.7.1. Polysaccharide Hydrolysis and Derivatization

RP-CrudeJSP, RP-JSP1, and RP-JSP2 fractions were analyzed for their characterization. Chitosan, starch, and chondroitin sulphate polysaccharides were treated as reference polysaccharides. Polysaccharide hydrolysis and derivatization was performed according to the method reported by Wu et al. [54], briefly modified. Polysaccharide samples (2 mg) were placed in 160 μL volume of TFA 4 M, in a sealed flask. After 15 min at room temperature, 40 μL of distilled water was added and samples incubated at 100 °C for 2 h, then 80 μL of distilled water was added and kept at 100 °C for 1 h more. The solutions were then cooled (rT) and centrifugated at 3000 rpm for 10 min (Hettich Zentrifugen MIKRO 120). The supernatants were dried at low pressure and washed several times with methanol, to completely remove the TFA. The derivatization was performed by dissolving the hydrolyzed samples in 320 μL of NH_3_ 7 N methanolic solutions. Aliquots of 100 μL were then transferred into clean tubes and 100 μL of 0.5 M PMP in methanol was added. The mixtures were allowed to react at 70 °C for 30 min, then cooled at rT and neutralized with 20 μL of 1% acetic acid. The mixtures were treated with equal volumes of water and chloroform (1 mL), shaken and chloroform layers were discarded. The washing was repeated at least three times. The aqueous samples were centrifugated at 10,000 rpm for 10 min and the supernatant stored at −20 °C until LC-MS analysis. 

#### 4.7.2. UHPLC-HR-ESI-Orbitrap/MS Analysis

Monosaccharide composition was determined on hydrolyzed and PMP derivatized samples by ultra-high-performance liquid chromatography (UHPLC) coupled to a high-resolution electrospray ionization source Orbitrap-based mass spectrometer (HR-ESI-Orbitrap/MS). The LC-MS system was composed by a Vanquish Flex Binary pump LC and an ESI Q Exactive Plus MS, Orbitrap-based FT-MS system (Thermo Fischer Scientific Inc., Darmstadt, Germany). The samples were diluted in methanol (1:2) and 5 µL each were injected on a C-18 Kinetex^®^ Biphenyl column (100 × 2.1 mm, 2.6 μm particle size) provided of a Security Guard TM Ultra Cartridge (Phenomenex, Bologna, Italy). The elution was performed at a flow rate 0.5 mL/min with formic acid in methanol 0.1% *v*/*v* (solvent A) and formic acid in H_2_O 0.1% *v*/*v* (solvent B) by using the following solvent gradient: 0–5.0 min, isocratic 35% A; 5.0–11.5 min, 35–53% A; 11.5–14.5 min, isocratic 53% A. The temperature of autosampler and column oven was maintained at 4 and 35 °C, respectively. HR mass spectra were acquired in both ESI negative and positive ionization modes (scan range *m*/*z* 400–800) operating in full (70,000 resolution, 220 ms maximum injection time) and data dependent-MS/MS scan (17,500 resolution, 60 ms maximum injection time) and optimizing ionization parameters as previously reported [55]. Hydrolyzed and PMP derivatized chitosan, starch, and chondroitin sulphate polysaccharides were analyzed in the same conditions and used as reference standards for the monosaccharide characterization.

### 4.8. Thermal Analysis

Thermogravimetric analysis (TGA) was carried out by using a Q500 instrument (TA Instruments, Milan, Italy) under a nitrogen flow of 60 mL·min^−1^, in the temperature range 30–700 °C and at a heating rate of 10 °C min^−1^. The sample weight and derivative weight as a function of temperature was recorded. The temperature of maximum degradation rate (Tmax) and the percentage weight residue at 700 °C were obtained.

Differential scanning calorimetry (DSC) analysis was carried out using a Mettler DSC-822 instrument (Mettler Toledo, Milan, Italy) under a nitrogen flow rate of 80 mL·min^−1^ and at a heating/cooling rate of 10 °C min^−1^, through a first heating cycle in the temperature range 30 to 150 °C to remove the adsorbed water by evaporation, and a second heating cycle from 30 to 250 °C. The glass transition temperature (Tg) was taken at the curve inflection point in the second heating cycle.

### 4.9. Biological Investigation

#### 4.9.1. Cell Culture Condition

Fibroblast BALB/3T3 clone A31 (CCL-163) and keratinocyte HaCat cell line were purchased from American Type Colture Collection (USA) and CellSystems (GmbH Germany), respectively. Cells were grown in a CO_2_ incubator at 37 °C and 5% CO_2_ with cells subcultured at 80–90% confluency, in Dulbecco’s Modified Eagle’s Medium (DMEM), supplemented with 2 mM L-glutamine and 1% penicillin/streptomycin) and 10% calf bovine serum (Merck, ITALY GERMAN). Cell monolayers were rinsed with PBS and treated with trypsin-EDTA to detach cells before resuspension in fresh media. 

#### 4.9.2. Cell Viability

Cell viability was conducted on either fibroblast s or keratinocytes. BALB/3T3 cells were seeded at a concentration of 10^4^ and 2.5 × 10^3^ cell per well for the analysis of cell viability at 24 and 48 h, respectively. HaCat cells were seeded in 96 well culture plate at a seeding density of 3 × 10^4^ and 2 × 10^4^ cells per well for the analysis of cell viability at 24 and 48 h, respectively. The cells were incubated at 37 °C in a 5% CO_2_ and left to proliferate for 24 h prior to the incubation with the samples. The culture medium from each well was removed and replaced with medium containing pre-dissolved extracted material in the range 0.01–2 mg/mL, in complete DMEM. After samples incubation (24 h or 48 h), media were removed and substituted with fresh medium containing 10% WST-1 reagent solution, for 4 h at 37 °C, 5% CO_2_. Afterwards, formazan dye absorbance was quantified at 450 nm with the reference wavelength of 655 nm by using multilabel reader (BioTek 800/TS, Thermo Scientific, Walthman, MA, USA).

#### 4.9.3. Scratch Test on BALB/3T3 Cells and HaCat Cells

The in vitro wound scratch test used in this study has already been described [10]. Briefly, BALB/3T3 cells were seeded, in 12-well plates, 1.25 × 10^5^ cells per well in DMEM medium containing 1% calf serum. Complete confluence was achieved after 24 h at 37 °C and 5% CO_2_. The HaCat cells were seeded in 12-well plates, 2.5 × 10^5^ cells per well in DMEM medium containing 1% FBS. Complete confluence was reached after 2 days at 37° C and 5% CO_2_. The resulting monolayers were scratched with a sterile pipette with p200 tip. After scratching, the wells of the plate were washed three times with PBS to remove debris and dead cells. Next, 2 mL of each sample was added at a concentration of 0.125 mg/mL per well. Culture medium was used as control. Micrographic images of each well with 4X objective (Nikon Eclipse Ts2R) were acquired by camera at time zero, after 4, 24 and 48 h. The acquired images were analyzed using the ImageJ Software (NIH, USA [56]) to determine the percentage of closure of the inflicted scratch (confluency rate) over time. The percentage was calculated according to:

confluency rate (%) = [(Area T0 − Area T)/Area T0] × 100(2)

where Area T_0_ is the area at time zero, Area T is the area at each endpoint. 

#### 4.9.4. Protection against Oxidative Damage

To evaluate the antioxidant activity of the RP-CrudeJSP, RP-JSP1 and RP-JSP2, the BALB/3T3 cells were subjected to oxidative stress. The cells were seeded in 96-well plates, at a concentration of 10^4^ cells per well and allowed to proliferate for 24 h at 37 °C and 5% CO_2_. After 24 h, the culture medium was removed, and cells were pre-treated with 0.125 mg/mL of extracted samples, for 2 h. Gallic acid 0.5 ug/mL was used as positive control, whereas complete DMEM medium kept for negative control samples. After washing with complete medium, the oxidative stress was provided by adding 1500 μM H_2_O_2_ and incubating for 1 h. Cell viability was evaluated by WST-1 assay and percentage of viability are referred to untreated, unstressed cells.

## 5. Conclusions

The extracted GAG-like polysaccharides stimulated the migration of cells, favoring wound closure in vitro, and displayed a significant protection versus induced oxidative stress. Despite the pristine RP-CrudeJSP was not endowed with antioxidative effect, it presents good cytocompatibility and wound healing potentials. Overall, the results suggest the use of RP-JSPs as functional excipients for epithelial tissue repair. Furthermore, the assessed chemical-physical features, the presence of net charges, the good thermal stability, as well as water solubility, are highly appreciated for their possible exploitation in the pharmaceutical and biomedical industry.

## Figures and Tables

**Table 1:** Characteristics of polysaccharides from *R. pulmo* jellyfish (RP-CrudeJSP, RP-JSP1 and RP-JSP2). Molecular weight (from Debye plot assay), protein content (from bicinchoninic acid assay), and weight percentage of sulphate groups (from gelatin—BaCl_2_ method).

Samples	Molecular Weight (kDa)	Protein % wt	Sulphate Groups % wt
RP-CrudeJSP	N.D. *	18.42 ± 0.71	24.20 ± 0.11
RP-JSP1	121 ± 6.33	25.13 ± 0.78	3.99 ± 0.22
RP-JSP2	590 ± 13.5	17.22 ± 0.42	25.92 ± 0.02

* N.D. not determined.

**Table 2:** UHPLC-HR-ESI-Orbitrap/MS data of monosaccharide detected in hydrolyzed and 1-phenyl-3-methyl-5-pyrazolone (PMP) derivatized polysaccharidse from *R. pulmo* jellyfish (RP-JSP1, RP-JSP2 and RP-CrudeJSP).

Peak ^a^	Compound	*t*_R_ (min)	HR- [M − H]^−^(*m*/*z*)	HR-MS/MS Product Ions (*m*/*z*) ^c^	Molecular Formula	Error (ppm)	Fraction
**1**	Glucosamine-PMP ^b^	5.4	508.2199	**214.10**, 173.07, 157.04	C_26_H_30_N_5_O_6_	−0.590	RP-JSP1, RP-JSP2, RP-CrudeJSP
**2**	Hexose1-PMP	7.4	509.2041	**215.08**, 173.07, 92.05	C_26_H_30_N_4_O_7_	−0.196	RP-CrudeJSP,
**3**	Galactosamine-PMP ^b^	7.9	508.2199	**214.10**, 173.07, 157.04	C_26_H_30_N_5_O_6_	−0.590	RP-JSP1, RP-JSP2, RP-CrudeJSP,
**4**	Hexose2-PMP	8.4	509.2041	**215.08**, 173.07, 92.05	C_26_H_30_N_4_O_7_	−0.196	RP-JSP1, RP-JSP2, RP-CrudeJSP,
**5**	Pentose1-PMP	8.8	479.1938	**215.08**, 173.07, 92.05	C_25_H_28_N_4_O_6_	+0.417	RP-CrudeJSP
**6**	Pentose2-PMP	9.3	479.1938	**215.08**, 173.07, 92.05	C_25_H_28_N_4_O_6_	+0.417	RP-JSP2
**7**	Deoxyhexose-PMP	9.6	493.2094	**215.08**, 173.07, 92.05	C_26_H_29_N_4_O_6_	+0.203	RP-JSP1
**8**	Pentose3-PMP	9.7	479.1938	**215.08**, 173.07, 92.05	C_25_H_28_N_4_O_6_	+0.417	RP-JSP1, RP-JSP2, RP-CrudeJSP
**9**	Glucose-PMP ^b^	12.2	509.2040	**215.08**, 173.07, 92.05	C_26_H_30_N_4_O_7_	−0.393	RP-JSP1, RP-JSP2, RP-CrudeJSP
**10**	Galactose-PMP ^b^	12.5	509.2040	**215.08**, 173.07, 92.05	C_26_H_30_N_4_O_7_	−0.393	RP-JSP1, RP-JSP2, RP-CrudeJSP
**11**	Pentose4-PMP	12.6	479.1938	**215.08**, 173.07, 92.05	C_25_H_28_N_4_O_6_	+0.417	RP-JSP1, RP-JSP2, RP-CrudeJSP

^a^ Compound numbers correspond with peak numbers in Figure 3. ^b^ Confirmed by reference standard. ^c^ The ion base peaks are shown in bold.

**Table 3:** Thermal parameters of polysaccharide from *R. pulmo* jellyfish (RP-JSP1, RP-JSP2 and RP-CrudeJSP). Temperature of the main degradation step (Tmax), residual weight percentage (Residue %), and glass transition temperature (Tg).

Sample	T_max_ (°C)	Residue (%)	T_g_ (°C)
RP-JSP1	285.1	20.9	81.9
RP-JSP2	313.5	44.3	88.8
RP-CrudeJSP	302.6	56.0	94.2

## Data Availability

Not applicable.

## Supplementary Materials

The following supporting information can be downloaded at: https://www.mdpi.com/article/10.3390/ijms231911491/s1.
