# Supplementary material for: Jellyfish Polysaccharides for Wound Healing Applications
Source: Int J Mol Sci. 2022 Sep 29;23(19):11491. doi: 10.3390/ijms231911491 (PMC9569628; doi:10.3390/ijms231911491)
Supplement: Supplementary file 1 [file ijms-23-11491-s001.zip › ijms-1948766-supplementary.pdf]

Supplementary information file of

Jellyfish polysaccharides for wound healing applications

Chiara Migone [1], Noemi Scacciati[1], Brunella Grassiri[1], Marinella De Leo[1,2], Alessandra Braca[1,2], Dario Puppi[3], Ylenia Zambito[1], and Anna Maria Piras [1,2,*]

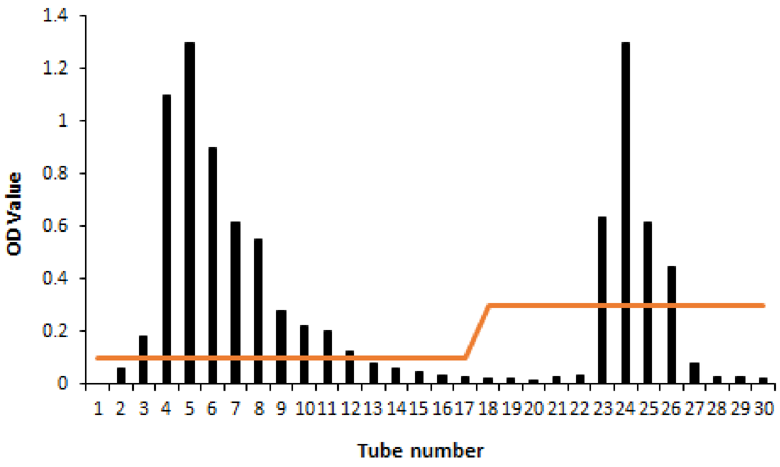

Figure S1. Elution curve of RP-Crude-JSP on a DEAE-sepharose column with water to NaCl 0.3 M elution (orange curve). Eluate was collected in tubes of 10 ml fractions each. Tubes 4-11 and 23-27 were collected for RP-JSP1 and RP-JSP2 fractions, respectively.

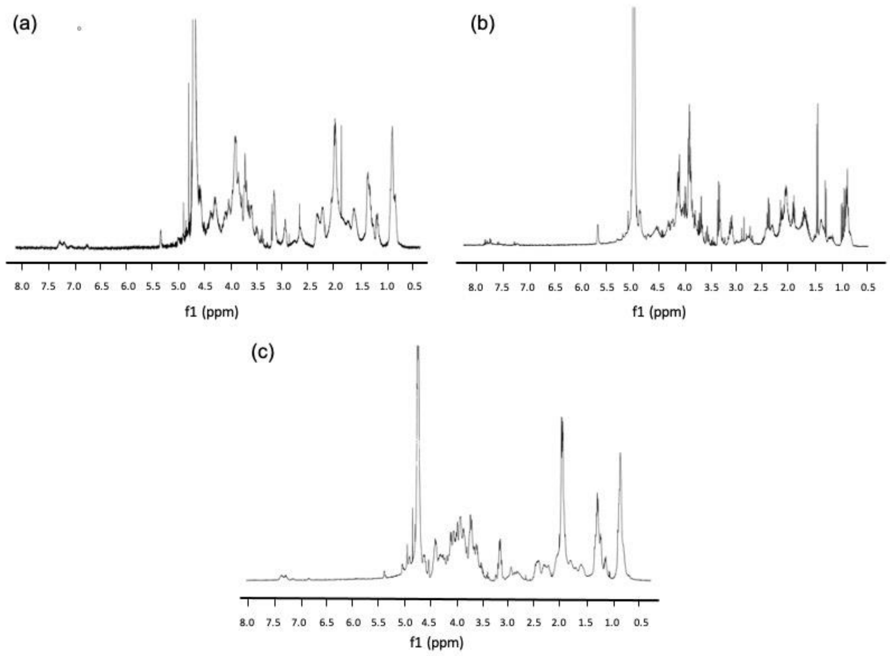

Figure S2. ^{1}H NMR spectroscopy in D_2O of **(a)** RP-CrudeJSP, **(b)** RP-JSP1 and **(c)** RP-JSP2.

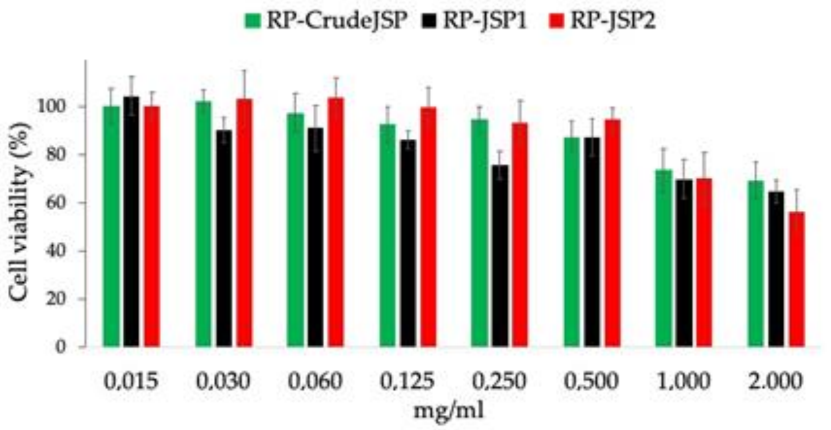

(a)

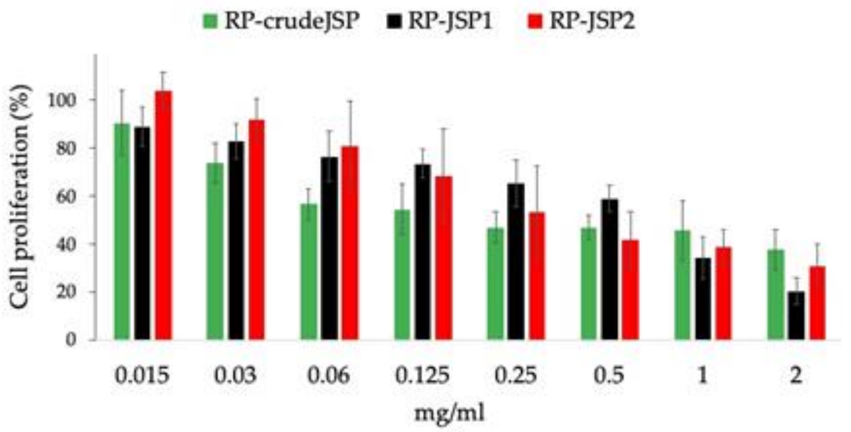

(b)

Figure S3. Cytotoxicity screening performed on BALB/3T3 cell line clone A31, exposed to RP-CrudeJSP, RP-JSP1 and RP-JSP2 in the 0.015–2 mg/ml concentration range: **(a)** cell viability after 24h of incubation; **(b)** cell proliferation after 48h of incubation. Untreated cells were used as control. The values indicated in the figure are means ± SD of 8 replicates.

Folin–Ciocâlteu method

RP-CrudeJSP, RP-JSP1 and RP-JSP2 were pre-solubilized in NaCl (0.3 M) and diluted with water until the final concentration of 0.125 mg/ml. The prepared samples were submitted to Folin-Ciocâlteau assay. Distilled water (1. 58 ml), 100 μl of Folin–Ciocâlteu reagent and 300 μl of Na_2CO_3 (20%) were added to 50 μl of samples. They were mixed and incubated in the dark at room temperature for 2 h. The absorbance was measured at 765 nm against a blank solution using a UV-VIS spectrophotometer. Gallic acid was employed as the standard in the concentration range 0.1-30 μg/ml ($R^2 = 0.999$). The samples were tested in quadruplicate.

RP-CrudeJSP, RP-JSP1 and RP-JSP2 did not show any reactivity under the assayed conditions, with ABS under the limit of detection and thus of the assay sensitivity. Concluding, at the tested sample concentration there is no species revealing gallic acid equivalent (GAE) reactivity.

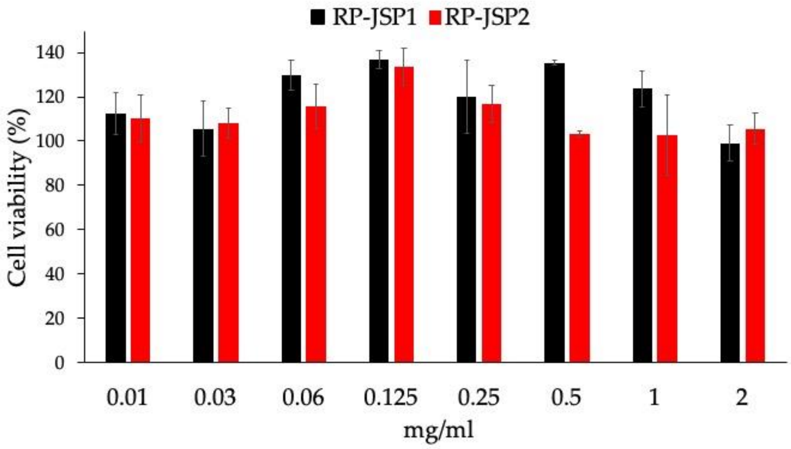

(a)

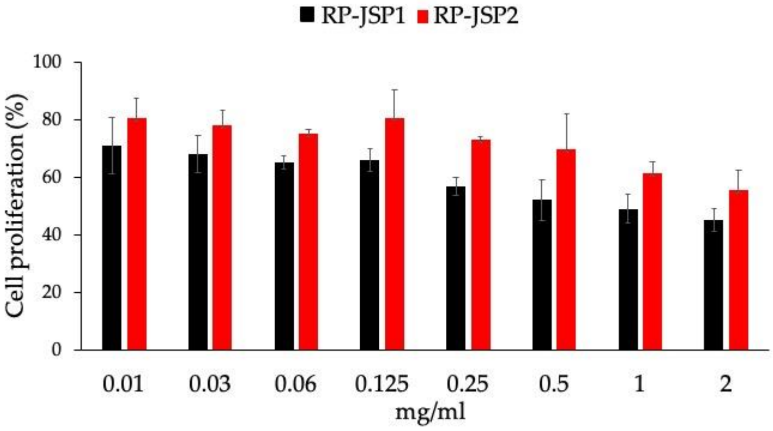

(b)

Figure S4. Cytotoxicity screening performed on HaCat cell line, exposed RP-JSP1 and RP-JSP2 in the 0.015–2 mg/ml concentration range: **(a)** cell viability after 24h of incubation; **(b)** cell proliferation after 48h of incubation. Untreated cells were used as control. The values indicated in the figure are means ± SD of 8 replicates.
